# Round the Hospitals

**Published:** 1920-02-28

**Authors:** 


### ROUND THE HOSPITALS.

The King held an Investiture in the Ballroom of Buckingham Palace on February 20, when the following members of the nursing services had the honour of receiving decorations from the hand of His Majesty:—Royal Red Cross and Bar: Miss Daisy Michell, Q.A.I.M.N.S. Royal Red Cross, First Class: Miss Mary Bonsor and Miss Hilda Drage, Q.A.I.M.N.S.; Miss Edith Davenport, Q.A.I.M.N.S. (R.); Miss Mary Dickinson, Miss Maud Dunn, Miss Margaret Newbould, Miss Edith Pilson, T.F.N.S.; Mrs. Mary Millar, C.N.S. Royal Red Cross, Second Class: Misses Isabelle Baron, Mabel Davis, Laura James (who also received the Military Medal), and Mary McNaughton, Q.A.I.M.N.S.; Mrs. Mary Binks, the Misses Julia Clancy, Bessie Dickson, Constance Eason, Martha

**ROUND THE HOSPITALS**—(*continued*).

Edge, Mary Smith, Grace Winter, Margaret Hilliard, and Mrs. Annie Townsend, Q.A.I.M.N.S. (R.); the Misses Helen Darge, Louise Dennis, Christina Davidson, Cecilia Lister, Shirley Wilson, and Mrs. Edith Mercer, T.F.N.S.; the Misses Joan O'Sullivan and Lillias Pumphrey, C.N.S.; the Misses Blanche Baldwin, May Broadbent, Amy Neale, Hilda Randolph, the Hon. Henrietta Mrs. Leith-Hay, Mrs. Hilda Llewellyn, B.R.C.S.; and Miss Lucy Hughes, V.A.D. His Majesty conferred the Military Medal on Miss Elizabeth Eckett, T.F.N.S., and Miss Selina Valentine, V.A.D. All the above ladies were received after the Investiture by Queen Alexandra at Marlborough House, when Dame Sidney Browne, R.R.C., was also present.

A LONG list of new awards of the Royal Red Cross by the King in recognition of valuable nursing services in connection with the war was issued on February 18:—Bar to the Royal Red Cross: Miss E. Holden, Miss J. Melrose, Miss F. N. Roberts, Miss E. A. M. Wilson. Royal Red Cross, First Class: Sister E. Bramwell; Miss A. Brooks, matron; Sister A. J. D. Durward; Assistant-Matron K. Fitton; Assistant-Matron M. Gregory; Matron E. H. Hay; Assistant-Matron A. Howard; Matron E. M. Humphries; Matron E. Hutchings; Assistant-Matron R. M. Rooke; Matron M. O. Sinzininex; Matron P. E. Smith; Matron A. H. Turnbull; Sister L. M. Wass; Matron E. White. All these ladies were already Associates of the Order. One hundred and seven matrons, sisters and nurses were admitted to the Second Class of the Royal Red Cross, together with one commandant and three members of Voluntary Aid Detachments.

WE learn that Dame Sidney Browne, G.B.E., Matron-in-Chief of the Territorial Force Nursing Service, whose impending retirement was announced some time ago, is definitely relinquishing her post on March 21 next. Dame Sidney's work for the Force and the country stands out even amidst all the great work done by medical and nursing administrators during the war. Her nursing record from the South African War onwards was briefly but tellingly depicted by Miss Cox-Davies at the complimentary dinner given to Dame Sidney last December, which was fully reported with a full-page illustration in our issue of December 13, 1919.

DAME SIDNEY BROWNE appeals through the medium of the *Daily Telegraph* for a rest-house for nurses disabled in the war. She says " a good many Army nurses who are permanently invalided from the Service have a very small pension granted to them—from £30 to £60 a year—and it is impossible for them to live on these pensions. Some of them have no homes. If any kind landowner would give a site for a small cottage for nurses on his estate we could build one or two cottages for them." Mrs. Martin Harvey writes:—" We shall shortly open a cottage home for nurses at Bonchurch, in the Isle of Wight. . . . It is my great ambition to have these cottage homes on the outskirts of every big provincial town as well as throughout Scotland. . . and I hope people will give houses for that purpose . . . we shall in the first place look after the nurses who have been 'broke' in the war." Nothing more forcibly illustrates the mass of unrelieved distress in the nursing profession than the rapidity with which homes founded for the relief of invalided nurses fill up and outrun their accommodation. There is room for all and more than all the generous endeavours now being made on behalf of the disabled among them.

THE inauguration of the forty-eight-hour week for nurses at St. George's Hospital is an event of the highest importance in the history of nursing. We believe St. George's to be the first London Hospital with a medical school attached which has accepted this great reform. The impetus which will thereby be given to the movement is incalculable. Twenty additional nurses have been appointed and have had accommodation provided for them at No. 3 Knightsbridge, a house formerly occupied by the French Red Cross. When objections are taken to the great additional cost of hospital nursing under these more humane conditions one very important consideration is ignored. The training of nurses is among the weighty benefits which the voluntary hospitals set out to procure for the public, and a system which extends the operations of the training-school, which secures a better class of women to be trained, and largely increases the number turned out every year ready for service is in no need of justification or apology. We trust St. George's will enjoy a record year in voluntary subscriptions as a token of public approval of their action.

THE Central Council for District Nursing in London holds its annual meeting on February 26 as this issue appears. The draft report for 1919 testifies to the increasing value of this unifying organisation in promoting the well-being of the affiliated Associations. As a distributing centre alone, for co-ordinating the bestowal of grants obtained from various public bodies, it more than justifies its existence. In addition it has done most valuable work in publishing and bringing up to date the admirable directory, in forming an emergency list of nurses, and in collecting information for the improvement of maternity nursing as distinct from midwifery. Fifty per cent. of doctors' cases are still nursed by untrained women; midwives are often over-worked and unable to give sufficient time to nurse their patients; doctors show an increasing distaste for undertaking midwifery, and there is need for more maternity hospitals. These facts were elicited from London Medical Officers of Health and reported at the informal conference on Maternity Nursing held under the ægis of the Central Council, which is devoting earnest consideration to the many difficult points connected with this matter.

**ROUND THE HOSPITALS—(*continued*).**

LARGELY due in the main to the generosity of Mr. C. R. Scrase Dickins, J.P., who is so well known in Brighton in connection with charitable work, a residential club for nurses, under the auspices of the Brighton and Hove Centre of the College of Nursing, Limited, was opened on February 21 by Sir Arthur Stanley, C.B., M.V.O. No better position than on the Marine Parade (Number 45) could possibly have been found, commanding as it does an unrivalled and uninterrupted view of the sea, and for seven years Mr. Scrase Dickins has loaned the premises rent free. There is accommodation for fifteen nurses, in addition to the resident staff, everything is up-to-date, and, above all, the scale of charges for residence is moderate. Sir Arthur Stanley congratulated the Centre upon having led the way in starting a residential club, mentioning that it was the first to be established, that Edinburgh and Dublin had followed the example, and that he hoped London would be equally favoured in the near future. The progress of the club will be followed with great interest, but with such names as Miss Kathleen Scott as Chairman of the Executive Committee, Miss A. M. Latham as Honorary Secretary and Treasurer, and Dr. Hobhouse as Chairman of the Finance Committee—names so long associated with the Royal Sussex County Hospital—there is little to fear but that the objects of the club will be fully realised. Captain Sir Thomas Butler, K.C.V.O., presided over the opening ceremony proceedings, which were also graced by the presence of the Mayor of Brighton (Alderman W. G. Wellman).

---

AN unpublished letter of Florence Nightingale is given in the February number of the *St. Thomas's Hospital Gazette*, written in 1858 to Dr. John Robertson, and now in the possession of his grandson, Dr. J. R. Carver. It contains a highly interesting comment on the rival systems of nursing organisation then struggling for the mastery. She says: "I deprecate equally the system when nurses, whether male or female, are under the command of the authorities of the hospital solely—in which case the arrangements as to hours, proprieties, sanitary things, etc., generally strike me as all but crazy. And [in] the system where the nurses, whether religious or secular . . . are in entire command of the hospital, the arrangements are generally equally crazy. . . . The collision (often disagreeable) between the secular administration and the nursing staff (whether nurses or nuns), as in the hospitals of London and Paris, is always salutary for the patients." This profound remark deserves to be pondered over. But nursing administrators ought to read the whole letter.

---

GREAT success has attended the efforts of the Home Service Ambulance Scheme launched last autumn by the Sussex branch of the B.R.C.S. The aim is to place a motor ambulance within a radius of not more than fifteen miles from every house in Sussex to effect the transport of all cases of accident and illness at a nominal scale of fees per mile, and especially to aid the Ministry of Pensions in the task of conveying disabled sailors and soldiers to hospitals where they can receive treatment. So far cars have been placed in West Sussex at Chichester, Storrington, and Horsham, while in East Sussex they are at Eastbourne, East Grinstead, and Hayward's Heath, with ambulance cars co-operating at Lewes and Brighton. The need is exemplified by the use made of the cars, as, for instance, at East Grinstead, in fourteen weeks, twenty patients were carried and 535 miles covered by the motor ambulance. It is much desired to form a centre in the east corner of the county whereby the admirable work done by the East Sussex Hospital in the districts round could be facilitated. We understand that at the Hastings Hospitals the authorities are most particular that all urgent cases should be admitted, even if makeshift accommodation has to be provided. It is under such good central organisation that an efficient ambulance service reaps its best fruits.

---

EMBLEY PARK, Florence Nightingale's former home is for sale. It is situated on the borders of the New Forest in magnificent woodland surroundings. The estate comprises 3,800 acres, and is noted for a rhododendron drive 2½ miles long. There are fine lakes on the property.

---

THE appeal of *The Spectator* for a convalescent home for Red Cross nurses has resulted in the offer of several houses to be let rent free for the purpose. That which proved most suitable and convenient was a large and beautiful country place, Tyrrells Wood, near Leatherhead. The house belongs to Major Keswick. It is large, well-appointed, and surrounded by delightful gardens and grounds, with a wide terrace facing Headley Downs. Major Keswick served in the Boer War and did good service for his country in the Great War. "There is always," says the Editor of *The Spectator*, "the sense of fellow service between the V.A.D. and the fighting man."

---

THE salaries of the nursing staff of the Royal Albert Edward Infirmary, Wigan, have been again increased; probationers being paid a scale of from £20 to £30 per annum are now to receive £5 yearly increments, and sisters £10 annual increments on their salaries of £50 to £70.

---

THE fund for the new Nurses' Home at the Great Northern Central Hospital is making good way. It is one of the objects for which the members of the League of Roses attached to the hospital collect money, and this League has collected over £7,300 since it was formed nine years ago to support the women's and children's wards. Princess Helena Victoria distributed badges and bars to the members of the League, of which she is President, on February 12, and the Marquis of Northampton presided on the occasion. It is always a good augury for the success of any undertaking such as this new Home

**ROUND THE HOSPITALS**—(*continued*).

when the nurses of the hospital themselves take the field. Sister Hill and Sister Aughton are to be congratulated on the success of their well-planned concert and variety entertainment on February 12 in the hall of the Northern Polytechnic, which has helped to bring the desired Home appreciably nearer.

The over-ambitious scheme formulated to co-ordinate the various nursing organisations in Wales and Monmouthshire has failed to secure sufficiently unanimous backing, and it has been decided to leave it in abeyance. The idea was to establish a Welsh National Committee to amalgamate in one central organisation all the county nursing associations, county councils, and boroughs with the general medical practitioners and whole-time medical officers, the Welsh Priory of the Order of St. John, and the British Red Cross Society. The difficulty of getting together representatives of these widely sundered societies and of setting up a constitution which should leave them free in their own spheres yet promote unity of action have proved insuperable. The time is evidently not ripe for this species of centralisation. The promoters have failed to make it clear what the constituent parts of the proposed central body stood to gain by adhesion, and general relief was experienced in all but one or two directions when the scheme fell to pieces by its own weight. What South Wales wants is not so much a new authority to add to the many now efficiently working, as *nurses*.

The Scarborough Nursing Association, which up to the beginning of last year undertook only general district nursing, has enlarged its scope at the instance of the Health Committee of the borough, which promised a grant, and has installed two midwives in their staff. The change necessitated the removal of the nurses' home to larger premises, but it has been successfully carried out. Sister Vickery, who has worked for some time under the Association, has been made Superintendent, and all is going smoothly, to the great satisfaction of the *clientèle*. There is great need for more subscribers, for the increased expenses have resulted in a deficit of £55 on the year's working.

Miss Skelling, matron of the Borough Infectious Hospital, Bangor, has contracted diphtheria in the course of her duties, and we understand that in other respects her health has suffered owing to her services in the institution. The City Council have voted her an honorarium of £10, with an expression of appreciation from the Mayor and Councillors. We should, we confess, be glad to be reassured as to the adequacy of her staff for the performance of their duties. Diphtheria is a risk all nurses have to encounter in infectious hospitals. But overwork is quite another thing, and the Council would do wisely to inquire into the number of hours on duty which led to Miss Skelling's breakdown.

The movement for bringing starving children from the famine areas to enjoy some of the blessings of the "happy English child" is already stirring many hearts to fruitful action. A Hospitality Committee has been formed with Major Haden Guest as the Chairman and temporary offices at 26 Golden Square, W. 1. There are many hundreds of thousands of children in Europe, suffering from rickets or other famine signs, and it is proposed to select from among them on the spot suitable candidates to be brought over here in Red Cross trains. They will be medically examined in their own country and again on arrival. They will first be provided with suitable clothing and from the reception camp they will be despatched to private houses for a year. Numbers of offers of free hospitality have been received from London, Liverpool, Newcastle, Sheffield, Bristol, and other places. It would be excellent work for the women's institutes in country villages to make themselves responsible for the keep of one child in their midst. Such deeds do more to promote the spirit of brotherhood among nations than many sermons.

The Royal United Hospital at Bath is among the zealous upholders of the modern professional spirit in nursing, and a well attended meeting took place on February 12 in the board-room of the hospital to hear an address from Miss G. Cowlin, the organising secretary of the College of Nursing. The Chairman of the hospital, Lieut.-Colonel E. Lewis, presided, and expressed himself a firm believer in the College. Miss Cowlin gave a succinct account of the College programme, including as it does far more than State registration. She was able to report that many training-schools for nurses, including that of the Royal United Hospital, Bath, were bringing their curriculum into line with the standards of the College, and congratulated the authorities on having already voluntarily adjusted their conditions so as to provide a broad professional training for probationers. Miss F. L. Mason, matron of the hospital, called attention to the *Daily Telegraph* Fund for endowing the nursing profession, and invited subscriptions from all present.

Principal A. H. Trow, speaking at a meeting of the Court of Governors on Thursday last week, gave many interesting details regarding the course which is to be established next October at the University College of South Wales and Monmouthshire for the training of health visitors. Under the scheme, which has been approved by the Board of Education, a university diploma will be established on a two years' course of training; the Board of Education is to make a grant of £20 per annum for each student who qualifies at the end of the course. Should the entry of students be considerable, as is anticipated, the scheme will be practically self-supporting. The course will be under the control of the Department of Preventive Medicine of the Welsh National Medical School.

